# Evaluation of Oral Health Status Based on the Decayed, Missing and Filled Teeth (DMFT) Index

**Published:** 2019-11

**Authors:** Ghobad MORADI, Amjad MOHAMADI BOLBANABAD, Ardavan MOINAFSHAR, Hemn ADABI, Mona SHARAFI, Bushra ZAREIE

**Affiliations:** 1. Social Determinants of Health Research Center, Research Institute for Health Development, Kurdistan University of Medical Sciences, Sanandaj, Iran; 2. Student Research Committee, Kurdistan University of Medical Sciences, Sanandaj, Iran

**Keywords:** DMFT index, Oral health, Adult, Epidemiology, Iran

## Abstract

**Background:**

The Decayed, Missing and Filled Teeth (DMFT) is a valuable index used for determining and monitoring the oral health status in a community. This study aimed to determine the oral health status and its associated factors based on the DMFT index among people aged 15 to 45 yr old in Kurdistan Province, west of Iran.

**Methods:**

This study was conducted on 2000 people aged 15–40 yr old in Kurdistan, western Iran in 2015. Using a questionnaire, data were collected by four trained dental students. The dependent variable was the DMFT index. The collected data were analyzed using T-test, ANOVA, Pearson statistics, Kendall statistics, and multiple regression.

**Results:**

The mean (SD) values of Decayed teeth (DT), Missing teeth (MT), and Filled teeth (FT) indices in the participants were 2.85±1.7, 1.15±1.84, and 3.33±1.7, respectively. The mean (SD) value of total DMFT index was 7.33±3.0. The results of multiple regression showed that the frequency of using dental floss (coefficient= –0.296, *P*=0.001), socio-economic status (coefficient=–0.199, *P*=0.001), parental education (coefficient= –0.183, *P*=0.001), frequency of brushing (coefficient=–0.182, *P*=0.001), and frequency of the use of mouthwash (coefficient=–0/143, *P*=0.001) had the highest level of with association with the DMFT index.

**Conclusion:**

The oral health status of the adult population is alarming and undesirable. The oral and dental health status can be improved via changing behavioral habits (such as brushing, using mouthwashes, and dental floss), promoting socioeconomic status, increasing individual’s and parent's level of education, and enhancing people’s access to health insurance.

## Introduction

Oral health is part of the general health that affects many of the daily activities of people, such as eating, talking, social relationships, and appearances (1, 2). The impact of oral and dental diseases on the quality of life is stronger than what seems to be (3, 4). Because of the significant effect of oral health on people's daily lives, WHO has identified oral health as one of the most important public health priorities in the world (5). Despite the great emphasis of WHO on oral health, it is still one of the public health problems, even in developed countries, and the problem is even more remarkable in developing countries (6). Even less-privileged people in Western European countries (with advanced dental care systems) have a poor dental health status (7). Overall, 60% to 90% of students and 100% of adults in the world have dental caries. In addition, about 30% of people aged 65 to 74 yr old in the world does not have any natural teeth (8).

For over 70 years, the Decayed, Missing and Filled Teeth (DMFT) index has been globally used as the most important index for assessing the status of oral and dental health. Moreover, this index is the most important index used in epidemiological studies of the health status of the community (9). This index determines the number of decayed teeth, the number of treated teeth, and the number of teeth missed due to decay (10). This index is used to evaluate and monitor oral health interventions in the community by developing policies and programs related to this area (11, 12).

Oral and dental health status of children and elderly, as the two high-risk groups, is continuously studied in most countries (13–17). Although dental caries have been a major problem for adult populations in developing and industrial countries over the past decade, dental caries and oral health status in adults have been less studied (18). In different studies in the world various methods have been used to investigate the prevalence of dental caries and the factors affecting it. The most important risk factors for dental caries were gender (19, 20), age (20, 21), level of education (19–22), oral and dental hygiene including brushing (20, 23, 24), oral and dental health literacy (23), and the economic status (19, 25).

The prevalence of dental caries is high in Iran (26). About 50% of 12-year-old children have dental caries (27). The results of a national oral health survey conducted in 2001 and 2002 showed that the mean DMFT score was 3.4 for young people aged 18 yr old and 11.0 for adults aged 35 to 45 yr old. In addition, 53% of the population aged 35 to 44 yr old had periodontal pockets (28). The population aged 15 to 45 yr old is economically the most productive population group in the community and improper oral health can affect their daily activities. Nevertheless, there is less data on the prevalence of dental caries and oral health status in this population group, as compared with children and elderly.

Therefore, this study aimed to investigate the oral health status and its effective factors based on the DMFT index among the people aged 15 to 45 yr old in Kurdistan Province.

## Methods

This study was a cross-sectional descriptive-analytic study conducted on people aged 15–40 yr old living in Sanandaj, Kurdistan, western Iran in 2015. The estimated sample size was 2200 people, and finally, the required data were collected from 2000 people. We used cluster sampling method and each cluster included 10 people who were in the desired age range. The heads of the clusters were selected based on the geographical encoding of Sanandaj, obtained from the Sanandaj post office. The required data were collected using a questionnaire and clinical examinations carried out through visiting the selected households.

The questioners were four trained dental students received the necessary training on how to complete the questionnaires and perform clinical examination. To calibrate between the four questioners, prior to the initiation of data collection process, each of the four trained dental students performed a survey on 25 subjects selected from among the study population; the mean stability between their findings was 97%. The questioners visited each household at their home and briefed the household members about the research project and then completed the questionnaire through asking questions about the following items: demographic information, insurance coverage status, socioeconomic status (SES), frequency of brushing during a day, frequency of the use of dental floss during a day, and frequency of the use of fluoride mouthwash during a day. In this study, the SES of individuals was determined through questioning about their assets, which is a more appropriate way in developing countries. Using the principal component analysis (PCA) method, the studied people were divided into five quintiles including poorest, poor, moderate rich, and richest (29).

The DMFT score of the samples were determined based on the results of clinical examination and calculation of the number of decayed (D), filled (F), and missed (M) teeth due to caries. The data were collected by questioners through observation and direct examination of the samples’ teeth using mirror number 4 and a medisporex catheter. During the examination, the subjects under examination and the researcher sat close to the window to perform the examination under the maximum natural light. After examining each patient, the results were recorded in the questionnaire.

In this study, the statistical analysis was performed using SPSS (ver.20, Chicago, IL, USA) software at a significance level of *P*<0.05. Descriptive statistics (Mean, Standard Deviation (SD), and frequency distribution tables) were used to describe the collected data. Using T-test and ANOVA, the DMFT index was assessed at different levels of the independent variables. The relationship between independent variables and the DMFT index was evaluated using Pearson statistics and Kendall statistics. The variables were entered into the multiple regression model for a *P*<0.05 in the univariate analysis. Finally, the variables that were significant in the regression test using a stepwise backward method, remained in the model.

The study was approved by the Ethics Committee of Kurdistan University of Medical Sciences (IR.MUK.REC.1393/1). All participants provided written informed consent before participating in the study.

## Results

Overall, 2000 adults aged 15–40 yr old were involved in this study, of whom 1039 people (51.8%) were male, 1012 people (50.6%) were married, and 1515 people (76%) had academic education. In addition, 80% of the respondents were under the coverage of health insurance, 88.2% had a household size fewer than four people, and 895 people (45%) were at the lowest socioeconomic group.

The mean (SD) values of DT, MT, and FT indices in the participants were 2.85±1.7, 1.15±1.8, and 3.3±1.7, respectively. The mean (SD) value of total DMFT index was 7.3±3.0 in all the participants, 6.9±2.8 in people aged 15–19 yr old, and 7.8±3.2 in people aged 35 to 45 yr old.

The DMF index was associated with the household size (*P*=0.008) and insurance status (*P*=0.008). This index is more unfavorable in adults with a household size of more than four (DMFT=7.8) and adults without insurance coverage (DMFT=8.2) (Table 1).

**Table 1:** Mean number of decayed, missing, and filled (D, M, and F) teeth and DMFT index by the demographic variables of respondents

***Variable ***		***Number (percentage)***	D Mean (SD^*^)	***M Mean (SD)***	***F Mean (SD)***	***DMFT Mean(SD)***	P-value^**^
Total population		2000		1.15 (1.8)	3.3 (1.7)	7.3 (3)	-
			2.85 (1.7)				
Sex	Man	1039 (52)	2.9 (1.6)	1.2 (2.1)	3.2 (1.7)	7.3 (3)	0.665
	Female	961 (48)	2.8 (1.8)	1.1 (1.5)	3.4 (1.7)	7.4 (3)	
Age groups	15–19	744 (37)	2.9 (1.7)	0.73 (1.2)	3.3 (1.6)	6.9 (2.8)	
	20–34	735 (37)	2.8 (1.7)	0.96 (1.4)	3.6 (1.9)	7.4 (2.9)	0.001
	35–45	521 (26)	2.8 (1.7)	2.02 (2.7)	3.01 (1.5)	7.8 (3.2)	
Marital status	Single	1012 (50.6)	2.9 (1.6)	0.72 (1.2)	3.2 (1.5)	6.8 (2.7)	
	Married	911 (45.5)	2.8 (1.7)	1.6 (2.2)	3.4 (1.8)	7.6 (3.2)	0.001
	Widow / widower	77 (3.9)	3.2 (2.0)	1.7 (2.4)	4.2 (2.5)	9.1 (2.9)	
Household size	4≤	1764 (88.2)	2.8 (1.7)	1.15 (1.8)	3.3 (1.6)	7.3 (3)	0.008
	> 4	238 (11.8)	2.9 (1.8)	1.2 (1.9)	3.7 (2.1)	7.8 (3.1)	
Insurance status	No	407 (20)	3.3 (2.0)	2 (2.4)	3.5 (2.3)	8.8 (3.15)	0.001
	Yes	1593 (80)	2.7 (1.6)	0.9 (1.6)	3.3 (1.5)	6.95 (2.8)	
Individual's education	Non-academic	485 (24)	3.1 (2.0)	1.7 (2.2)	3.3 (2.1)	8.1 (3.38)	
	Academic	1515 (76)	2.76 (1.6)	1 (1.7)	3.4 (1.55)	7.1 (2.8)	0.001
Parental education	Non-academic	516 (26)	3.25 (1.97)	2.2 (2.3)	3.8 (2.5)	9.3 (3.1)	0.001
	Academic	1484 (74)	2.7 (1.6)	0.8 (1.5)	3.2 (1.3)	6.65 (2.7)	
Socio-economic class (SES)	Poorest	895 (45)	3.5 (1.8)	1.9 (2.3)	3.5 (2)	8.9 (2.8)	
	Poor	370 (18)	2.7 (1.2)	0.6 (1.4)	3.5 (1.3)	6.9 (2.1)	
	Moderate	282 (14)	2.5 (1.3)	0.5 (0.9)	3.2 (1.4)	6.2 (2.6)	0.001
	Rich	241 (12)	2.0 (1.1)	0.4 (0.7)	3.0 (1.5)	5.4 (2.4)	
	Richest	212 (11)	1.75 (1.5)	0.5 (0.8)	2.9 (1.4)	5.2 (3)	

* SD: Standard Deviation //

** *P*-value is related to difference subgroups for DMFT

The results of ANOVA showed that the DMFT index was associated with age group (*P*=0.001), marital status (*P*=0.00), Individual's education (*P*=0.001), parental education (*P*=0.001), and socio-economic class (*P*=0.001). This index was more unfavorable in people aged 35 to 45 yr old (DMFT=7.83), widows and widowers (DMFT=9.05), people with a non-academic education level (DMFT=8.1), people with a non-academic parental education (DMFT=9.3), and people in the poorest social class (DMFT=8.9) (Table 1).

As shown in Table 2, 1148 (57%) of the participants reported that they were brushing their teeth once a day. In addition, 54% and 85.7% of the subjects, respectively, reported that they were not using dental floss and mouthwash daily.

**Table 2:** Mean number of decayed, missing, and filled (D, M, F) teeth and DMF index by factors practiced to observe oral and dental health

***Variable***		***Number (percentage)***	D Mean (SD^*^)	***M Mean (SD)***	***F Mean (SD)***	***DMFTMean (SD)***	P-value^**^
Frequency of tooth brushing per day	0	79 (4)	4.0 (2.9)	5.2 (4.00)	1.9 (2.1)	11.1 (3.4)	
	1	1148 (57)	3.2 (1.7)	1.2 (1.6)	3.5 (1.8)	7.8 (2.9)	0.001
	≥ 2	773 (39)	2.3 (1.3)	0.7 (1.3)	3.2 (1.4)	6.2 (2.5)	
Frequency of dental flossing per day	0	1098 (55)	3.5 (1.7)	1.6 (2.2)	3.4 (1.7)	8.85 (2.8)	
	1	831 (41)	2.2 (1.3)	0.6 (0.9)	3.3 (1.7)	6 (2.5)	0.001
	≥ 2	71 (4)	1.3 (1.5)	0.5 (0.9)	2.2 (1.3)	4.0 (2.7)	
Frequency of using mouthwash per day	0	1713 (85.7)	3.0 (1.7)	1.3 (1.9)	3.4 (1.7)	7.7 (2.9)	
	1	224 (11.2)	1.8 (1.4)	0.5 (0.9)	2.9 (1.3)	5.3 (2.6)	0.001
	≥ 2	63 (3.2)	1.4 (0.9)	0.2 (0.4)	2.3 (1.3)	3.8 (1.8)	

* SD: Standard Deviation //

** *P*-value is related to difference subgroups for DMFT

The results of the ANOVA showed that the DMFT index was associated with the frequency of brushing daily (*P*=0.001), frequency of the use of dental floss daily (*P*=0.001), and frequency of the use of mouthwash daily (*P*=0.001). This index was more unfavorable in people who did not brush their teeth (DMFT=11.1), did not use dental floss (DMFT=8.85), and did not use mouth-wash (DMFT=7.7). The variables in Tables 1 and 2 which had a significant relationship with the DMFT index were entered into the regression model as covariates. The model with the highest R2 was determined by backward method (R2=0.44); the results are shown in Table 3.

**Table 3:** Backward regression model for predicting DMFT index

***Variable***	***B (coefficient)***	***Std.B ***	***Beta ***	***T ***	**P*-value***	95%CI^*^ for (B)
Constant	11.27	0.311	-	36.23	0.001	10.66 , 11.88
Parental education	−1.26	0.138	−0.183	−9.15	0.001	−1.53 , −0.99
Individual’s education	0.25	0.127	0.036	2.006	0.045	0.006, 0.5
Socioeconomic status	−0/43	0/043	−0.199	−9.93	0.001	−0.54, −0.31
Marital status	0.48	0.119	0.092	4.058	0.001	0.24, 0.71
Insurance status	−0.35	0.14	−0.047	−2.53	0.012	−0.62, −0.07
Age group	0.31	0.086	0.081	3.56	0.001	0.14, 0.48
Frequency of tooth brushing	−0.99	0.098	−0.182	−10.141	0.001	−1.18, −0.8
Frequency of using mouthwash	−0.94	0.118	−0.143	−8.011	0.001	−1.17, −0.71
Frequency of using dental floss	−1.57	0.098	−0.296	−16.125	0.001	−1.76, −1.38

* Confidence interval (CI)

The frequency of using dental floss (coefficient= –1.57, 95% confidence interval (CI) –1.76, –1.38), Parental education (coefficient= –1.26, 95% CI –1.53, –0.99), Frequency of tooth brushing (coefficient= –0.99, 95% CI –1.18, –0.8), Frequency of using mouthwash (coefficient = –0.94, 95% CI –1.17, –0.71) had the highest level of association with the DMFT index. The other effective variables were socioeconomic status, marital status, age group, insurance status, and Individual's education.

## Discussion

The DMFT index was 7.3 in all the participants, 6.9 in people aged 15–19 yr old, and 7.8 in people aged 35 to 45 yr old, which is unfavorable according to WHO (30). Probably, the reasons are the inadequate attention of families to oral health, low financial accessibility due to uncovering such services in insurance programs, and insufficient government attention to community-based oral health promotion programs. The DMFT index in people aged 35 to 45 yr old was 14.8 in Iran, 12.28 in Japan, 12.10 in Malaysia, 5.2 in South Korea, and 10.8 in Turkey (17). The DMFT index in people aged 35 to 45 yr old was 4.10 in Iran, 3.24 in Japan, 2.9 in Malaysia, 3.57 in South Korea, and 2.3 in Turkey (7). The data about these countries were obtained from surveys performed during 2002 to 2010, so it is difficult to compare them with each other.

In a study conducted on Iranians aged 35 to 45 yr in all parts of Iran, the DMFT index was reported to be 11.00 ± 6.4 (28). The results on people aged over 30 yr are different from our findings; this difference might to be attributed to the increase in people’s access (physical, financial, and cultural access) to oral health care which has led to an improvement in the economic status of the people and increased their mean education level over the last decades. Moreover, the difference might be also attributed to interventions by The Ministry of Health and Medical Education (MOHME) of Iran such as the use of sodium fluoride mouthwash for elementary students (6 to 12 yr old) with a mean coverage of 90%, and the provision of varnish floor therapy for elementary students (6 to 14 yr old) with a mean coverage of 83% on a regular basis, twice a year (31).

Frequency of the use of dental floss, socioeconomic status, parental education, frequency of brushing, frequency of the use of mouthwash, marital status, age group, insurance status, and individual's education were the factors influencing DMFT index.

Individuals with an academic education degree had a better mean DMFT score. Individuals or parental education has always been one of the variables influencing individual health. Academic education is associated with improving economic status n and health literacy; as a result, it is necessary to improve financial and cultural access to oral care services. In Mexico, poor dental health status was associated with lower level of education (32). The relationship between lower education level and adverse health status has also been reported (19–22).

People without health insurance coverage had poorer oral and dental health status. The odds ratio of higher DMFT index in non-insured individuals was 20% to 40% more than that in people with a health insurance coverage (33). Iran health insurance does not cover oral care services (34). However, insurance coverage helps to save the household's costs in other health care fields, as a result, it somewhat preserves people’s financial funds to ask for oral care services.

In the present study, people with poorest SES had more unfavorable DMFT index. In many studies, the relationship between poor economic status and poor oral health is reported (35, 33, 19). Probably a large part of the lack of financial access to health care services is due to health insurance scheme’s poor coverage or lack of coverage for oral health care services.

The findings of this study showed that as age increases, the DMFT index becomes more unfavorable. With aging, the number of decayed, missed, and filled teeth normally increases; thus, the WHO sets a higher DMFT index for people of polder ages (30). With increasing the age, the DMFT index was higher (36). In addition, some other studies have reported the relationship between older age and poorer oral health status (20, 21).

The results of our study indicated that brushing, using dental floss, and using oral mouthwash improved the DMFT index. Many studies have reported the positive effects of good oral health habits (including those mentioned above) on oral and dental health (20, 24, 37, 38). Inappropriate oral health habits increases the incidence of oral infections and may lead to more unfavorable oral health status (39). Furthermore, behavioral habits such as brushing, using mouthwashes and floss, having a balanced diet, and regular referral to dentists are associated with reduced risk of tooth decay (40).

As one of the limitations of this study, it was conducted only in a city which was due to financial and logistical constraints. In addition, because of the same constraints, we were not able to cover all age groups, which is another limitation of this study.

## Conclusion

Despite the interventions carried out in recent years, oral and dental health in the adult population is alarming and inappropriate. Oral and dental health status can be improved via changing behavioral habits (such as brushing, using mouthwashes, and dental floss), promoting socioeconomic status, increasing individual’s and parental education, and enhancing people’s access to health insurance. When implementing dental caries prevention programs, it is necessary to pay special attention to people with lower socioeconomic status as they have more unfavorable behavioral habits and lower education level.

## Ethical considerations

Ethical issues (Including plagiarism, informed consent, misconduct, data fabrication and/or falsification, double publication and/or submission, redundancy, etc.) have been completely observed by the authors.
